# Bronchoscopic local steroid spray to prevent bronchial tuberculosis-induced cicatricial bronchial stenosis: A case series

**DOI:** 10.1097/MD.0000000000011464

**Published:** 2018-07-13

**Authors:** Daizo Yaguchi, Motoshi Ichikawa, Masato Shizu, Noriko Inoue, Daisuke Kobayashi, Naoyuki Imai

**Affiliations:** Deparment of Repiratory Medicine, Gifu Prefectural Tajimi Hospital, Tajimi, Gifu, Japan.

**Keywords:** bronchoscopy, endobronchial tuberculosis, local steroid spray, tuberculous bronchial stenosis

## Abstract

**Ratonale:**

Cicatricial bronchial stenosis or obstruction occurring in the healing process of endobronchial tuberculosis (ET) is a problematic complication of tuberculous airway lesions. Prevention by internal medical treatment is desired.

**Patient concerns:**

This case series describes four patients who diagnosed ET with Type IIIb (protruding ulcer-type) based on Arai's classification of bronchoscopic findings of bronchial tuberculosis.

**Diagnoses:**

Endobronchial tuberculosis.

**Interventions:**

A local steroid spray was applied bronchoscopically to active protruding ulcer-type lesions (which are likely to cause cicatricial stenosis) that extended in the transverse direction and occupied one-half or more of the circumference on bronchoscopy.

**Outcomes:**

Cicatricial stenosis was prevented in two of four patients. Treatment was discontinued in athird patient because tolerance could not be achieved, although the patient's condition had improved. In the fourth patient, treatment was switched to systemic steroid administration because of a problem with tolerance and the broad range of the lesion; however, stenosis remained.

**Lessons:**

Local steroid spray-applied bronchoscopically to bronchial tuberculosis lesions in the ulcer formation and granulation periods may help prevent stenosis.

## 1 Introduction

Cicatricial bronchial stenosis or obstruction occurring in the healing process of endobronchial tuberculosis (ET) is a problematic complication of tuberculous airway lesions.^[1]^ Its prevention by internal medical treatment without invasive treatment such as intervention by stenting and balloon dilatation^[2]^ or surgical treatment^[3]^ is desired.

In our hospital, steroid drugs are systemically administered to selected patients as an adjuvant therapy to prevent cicatricial bronchial stenosis^[4]^; however, no standardized method has been established. Moreover, systemic steroid administration should be avoided as much as possible because it frequently causes adverse reactions such as inducing immunosuppression. The objective of the present treatment is to prevent tuberculous cicatricial bronchial stenosis and obstruction as much as possible by administering a local steroid to lesions and thereby prevent the later need for invasive treatment.

## 2 Patients and methods

By using Arai's classification of bronchoscopic findings of bronchial tuberculosis,^[5]^ we selected Type IIIb (protruding ulcer-type) lesions (which are likely to subsequently cause cicatricial stenosis) in whom the active lesions extended in the transverse direction and occupied one-half or more of the circumference on bronchoscopy. The patients received a local spray with 400 μg of bronchoscopic fluticasone propionate nose drops or 10 mg of triamcinolone acetonide under bronchoscopy. The dose of the drug was set at the maximum dose covered by the Japanese National Health Insurance as indicated in the package insert. Since a bronchoscopic procedure once every 1 to 2 weeks was acceptable to the patients, the frequency of procedure used a similar time frame. Treatment was repeated every 1 to 2 weeks depending on the patient's condition, and the criterion to complete treatment was set at either regression/disappearing of the target ulcerative lesion (Type IIIb) to <1/2 of the circumference of the lumen or transition to the granulation type (Type IV) on bronchoscopy based on Arai's classification of bronchoscopic findings of bronchial tuberculosis. Observation of the lumen by bronchoscopy and evaluation by CT at least once within approximately 6 months to 1 year after completion of treatment were set as the time frame and method of follow-up. Either sputum or a specimen collected under bronchoscopy was periodically submitted for the *Mycobacterium tuberculosis* (*M tuberculosis*) culture test, and the time to negative conversion of the samples in culture was confirmed. Our study is a retrospective study. Written informed consents were obtained from the patients for publication of this manuscript and any accompanying images and our institutional review board approved the study protocol.

## 3 Results

The characteristics of the 4 patients and results are shown in Table 1. Cicatrical stenosis was prevented in 2 of the 4 patients. Although 1 of the 2 patients transferred to another hospital, the white coat had regressed to <1/2 of the circumference, suggesting that prevention of cicatrical stenosis in the future can be expected. However, cicatrical stenosis could not be prevented in the other patient.

**Table 1:**
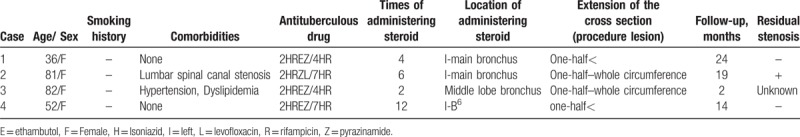
The characteristics of the 4 patients and results.

### 3.1 Case 1

**Figure 1:**
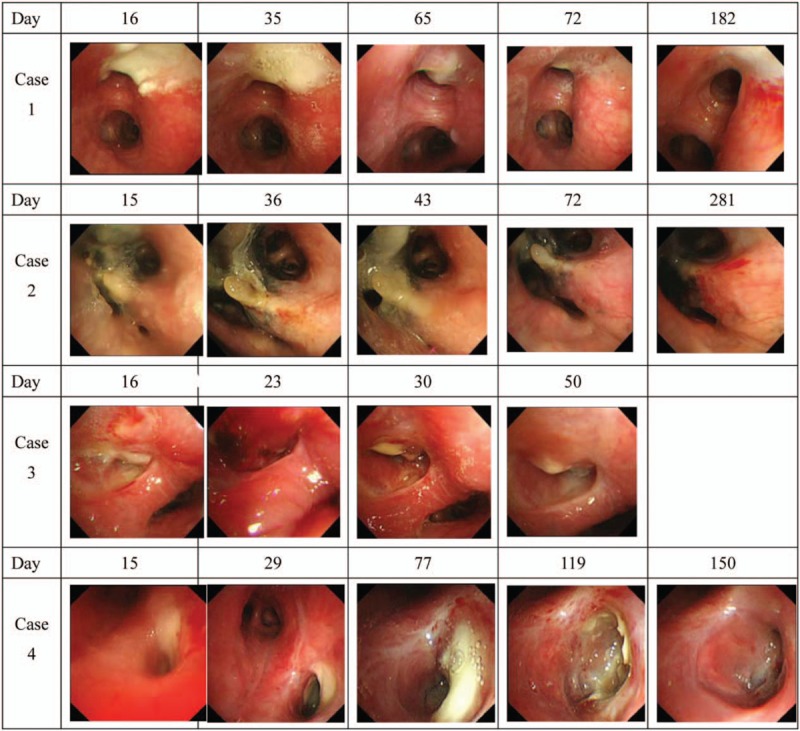
Time-course changes in bronchoscopic findings of the lesions treated with a local steroid spray in Cases 1–4.

**Figure 2:**
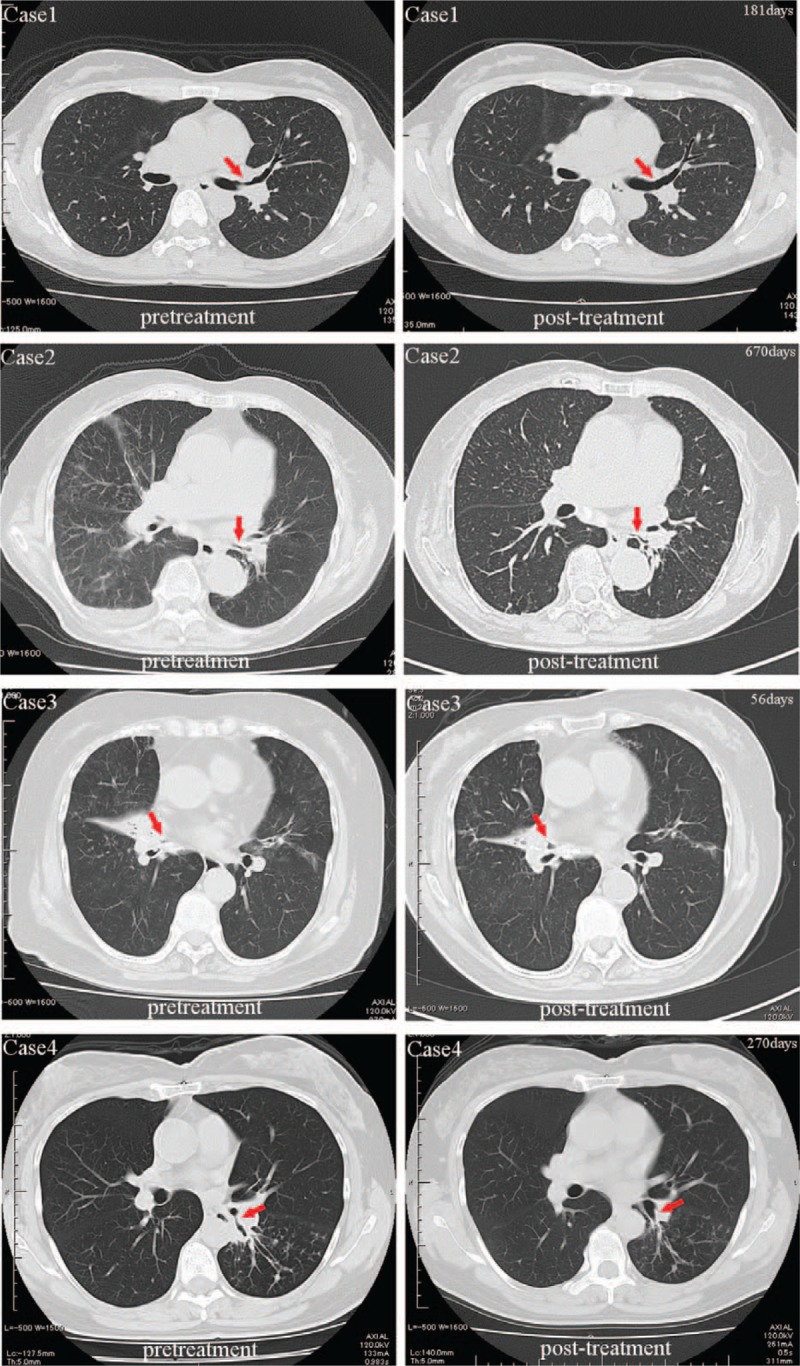
Chest computed tomography (CT) images before and after treatment in Cases 1–4. The figure in the right upper corner of each post-treatment CT image indicates the time (number of days) from the initiation of the administration an antituberculous drug. The arrows indicate the local steroid spray-applied airway regions. CT = computed tomography.

The patient was a 36-year-old woman. A dry cough had persisted for approximately 2 months, and her physician had treated her with oral antibiotics, but her condition did not improve. She then visited our hospital. The results of acid-fast bacterium smear and PCR for *M tuberculosis* were both positive. Lesions with a stenosis rate of 25% to 50%, based on the airway stenosis classification described by Freitag et al,^[6]^ were noted primarily in the left main bronchus. A local spray with fluticasone propionate nasal drops was administered for 1 week, 4 times in total and follow-up time period was 24months. Cicatricial stenosis was successfully prevented (Figs. 1 and 2).

### 3.2 Case 2

The patient was an 81-year-old woman. She visited a physician for complaints of cough and fever persisting for several days. On radiography, pneumonia was suspected and treated; however, her symptoms did not improve. The smear and polymerase chain reaction (PCR) results for *M tuberculosis* were both positive; therefore, she was transferred to our hospital. Lesions with a stenosis rate of approximately 50% were primarily in the left main bronchus, and local steroid spray was administered for 1 week, 6 times in total and follow-up time period was19 months. This protocol was similar to that in Case 1. There was improving a narrowing of the bronchial lumen by the ulceration of the protruding granulation covered with a white coat, but she could not tolerate bronchoscopy and the lesion extended. Thus, treatment was switched to systemic steroid administration; however, cicatricial stenosis eventually remained (Figs. 1 and 2).

### 3.3 Case 3

The patient was an 82-year-old woman. A dry cough had persisted for approximately 3 months and bloody sputum appeared. Therefore, she visited a physician. The results of acid-fast bacterium smear and PCR for *M tuberculosis* were both positive; therefore, she was transferred to our hospital. A lesion with a stenosis rate of 90% was at the entrance of the middle lobar bronchus. Local spray with triamcinolone acetonide was administered for 1 week, 2 times in total and follow-up time period was 2 months. There was improving a narrowing of the bronchial lumen by the ulceration of the protruding granulation covered with a white coat, but she could not tolerate bronchoscopy. The treatment was completed. She transferred to another hospital because her home was distant (Figs. 1 and 2).

### 3.4 Case 4

The patient was a 52-two-year-old woman. A wet cough had persisted for approximately 1 month and a physician treated her with antibiotics. However, her condition did not improve. Results of the acid-fast bacterium smear and PCR for *M tuberculosis* were both positive. She was referred to our hospital. Lesions with a stenosis rate of 25% to 50% were primarily at the entrance of the left B6. Local steroid spray was administered for 1 to 2 weeks, 12 times in total and follow-up time period was 14months. This protocol was similar to that in Case 3. Stenosis improved. Cicatricial stenosis was prevented (Figs. 1 and 2).

## 4 Discussion

In Japan, the frequency of tracheobronchial tuberculosis complications in patients with pulmonary tuberculosis was 10% to 20% in the 1950s and has decreased to 2% in these days.^[7]^ Its frequency in Turkey and Korea are 2%^[8]^ and 54%,^[9]^ respectively, which demonstrates its variability among medical areas.

The definition of tracheobronchial tuberculosis is tuberculosis lesions in the trachea and central bronchi in the upper rather than the segmental bronchi,^[10]^ which was the previous definition. This study followed the current definition.

How to prevent cicatricial bronchial stenosis and obstruction during the healing process of bronchial tuberculosis has been discussed, and various adjuvant therapies have been attempted^[4,11]^; however, no treatment has been established. Once the complication of cicatricial bronchial stenosis or obstruction occurs and clinical symptoms appear such as recurrent pneumonia and dyspnea, then invasive treatment options such as intervention by stenting and balloon dilatation^[2]^ and surgical treatment^[3]^ can place a heavy burden on patients. The treatment in the present study was a noninvasive method aimed at preventing cicatricial bronchial stenosis or obstruction. Prevention could not be achieved and stenosis unfortunately developed in one patient owing to complex conditions caused by problems tolerating bronchoscopy and the longitudinally extending wide range of the lesion. However, prevention have been achieved in 2 of the other 3 patients. The patency of the entrance of the bronchus and recession of the ulcerative lesion were observed after only 2 administrations in the one patient, which suggests that if she could have tolerated bronchoscopy, stenosis would have been sufficiently prevented.

For the target of treatment, based on Arai's classification of bronchoscopic findings of bronchial tuberculosis,^[5]^ we selected patients with Type IIIb (protruding ulcer-type) lesions (which are likely to cause cicatricial stenosis) in whom active lesions extended in the transverse direction and occupied one-half or more of the circumference on bronchoscopy. The transverse extension and the depth of lesions (i.e., the grade of destruction of the smooth muscle layer and cartilage, which are the supporting tissue) may subsequently lead to stenosis or obstruction as fibrosis (i.e., cicatrization) of the lesion progresses, and lesions with transverse extension exceeding one-half of the circumference may later induce stenosis or obstruction accompanied by deformity.^[12]^ The distance of advancement of lesions (20 mm or longer) and reduction of the forced expiratory volume in 1 second (i.e., <80%) at the time of treatment initiation are other stenosis-predictive factors.^[9]^ A tuberculous cicatricial bronchial stenosis/obstruction-predicting score classification by combining these factors will be needed in the future.

The bacterial negative conversion period did not markedly differ among the patients. The effect of local steroid injection against benign airway stenosis has been reported.^[13,14]^ Based on the finding that a local spray was an effective treatment, we are planning to investigate improvement of the effect by the administration of not only a local spray but also local injection of steroid to prevent cicatricial bronchial stenosis and obstruction. When the patient coughs during the treatment which can often happen, the sprayed drug solution may not efficiently infiltrate the lesion. Thus, we believe that injection is more effective than spray. So that, we have investigated and performed local steroid injection as an efficient and effective method of treatment (This trial was registered in the university medical information network. Registration date: July 31, 2017; UMIN000028457).

## 5 Conclusions

Local steroid spray may help prevent stenosis caused by bronchial tuberculosis in the ulceration and granulation periods. The desirable indications for local treatment, appropriate dose for local spray, the possibility of administering a local injection, the evaluation of the depth by echography, and tolerability of patients to bronchoscopy are subjects that need to be investigated. Some patients require intervention and surgical treatment, even though they receive adjuvant therapy. We will select and accumulate cases for which invasive treatment can be avoided by administering a local steroid. For future treatments, we are now performing a prospective pilot study employing steroid injection instead of a spray to prevent tuberculous bronchial stenosis as bronchoscopic local steroid administration (UMIN000028457).

## Author contributions

**Data curation:** Daizo Yaguchi.

**Investigation:** Daizo Yaguchi, Masato Shizu.

**Methodology:** Daizo Yaguchi.

**Project administration:** Motoshi Ichikawa, Masato Shizu, Noriko Inoue, Daisuke Kobayashi, Naoyuki Imai.

**Writing – original draft:** Daizo Yaguchi.

**Writing – review & editing:** Daizo Yaguchi, Motoshi Ichikawa.
